# Potentiation of NMDA Receptor-Dependent Cell Responses by Extracellular High Mobility Group Box 1 Protein

**DOI:** 10.1371/journal.pone.0044518

**Published:** 2012-08-31

**Authors:** Marco Pedrazzi, Monica Averna, Bianca Sparatore, Mauro Patrone, Franca Salamino, Manuela Marcoli, Guido Maura, Chiara Cervetto, Daniela Frattaroli, Sandro Pontremoli, Edon Melloni

**Affiliations:** 1 Department of Experimental Medicine (DIMES) University of Genova, Genova, Italy; 2 Centre of Excellence for Biomedical Research (CEBR), University of Genova, Genova, Italy; 3 Dipartimento di Scienze e Innovazione Tecnologica, DiSIT, University of Piemonte Orientale “Amedeo Avogadro”, Alessandria, Italy; Univ. Kentucky, United States of America

## Abstract

**Background:**

Extracellular high mobility group box 1 (HMGB1) protein can operate in a synergistic fashion with different signal molecules promoting an increase of cell Ca^2+^ influx. However, the mechanisms responsible for this effect of HMGB1 are still unknown.

**Principal Findings:**

Here we demonstrate that, at concentrations of agonist per se ineffective, HMGB1 potentiates the activation of the ionotropic glutamate N-methyl-D-aspartate receptor (NMDAR) in isolated hippocampal nerve terminals and in a neuroblastoma cell line. This effect was abolished by the NMDA channel blocker MK-801. The HMGB1-facilitated NMDAR opening was followed by activation of the Ca^2+^-dependent enzymes calpain and nitric oxide synthase in neuroblastoma cells, resulting in an increased production of NO, a consequent enhanced cell motility, and onset of morphological differentiation. We have also identified NMDAR as the mediator of HMGB1-stimulated murine erythroleukemia cell differentiation, induced by hexamethylenebisacetamide. The potentiation of NMDAR activation involved a peptide of HMGB1 located in the B box at the amino acids 130–139. This HMGB1 fragment did not overlap with binding sites for other cell surface receptors of HMGB1, such as the advanced glycation end products or the Toll-like receptor 4. Moreover, in a competition assay, the HMGB1_(130–139)_ peptide displaced the NMDAR/HMGB1 interaction, suggesting that it comprised the molecular and functional site of HMGB1 regulating the NMDA receptor complex.

**Conclusion:**

We propose that the multifunctional cytokine-like molecule HMGB1 released by activated, stressed, and damaged or necrotic cells can facilitate NMDAR-mediated cell responses, both in the central nervous system and in peripheral tissues, independently of other known cell surface receptors for HMGB1.

## Introduction

The different effects exerted by extracellular HMGB1 on specific target cells have been related to the ability of this protein to interact with alternative cell surface receptors, such as the receptor for advanced glycation end products (RAGE) and the toll-like receptors (TLRs) 2 and 4 [1], [2]. However, a number of observations indicate that HMGB1 can also act as a co-stimulating accessory protein without association with these receptors [3]–[5]. In this respect, we have demonstrated previously that HMGB1 administered to erythroleukemia cells, in the presence of hexamethylenebisacetamide, or to epidermoid carcinoma cells, in the presence of epidermal growth factor, induced an increased cell calcium influx leading to activation of erythroid differentiation or cell motility, respectively [6], [7]. Although we excluded the involvement of RAGE in these cell responses to HMGB1, the receptor responsible for the elevation of [Ca^2+^]_i_ promoted by HMGB1 was not identified [8]. Recently, it has been demonstrated that HMGB1-TLR4 signaling activates the Ca^2+^ conductance of the glutamate gated NMDAR contributing to acute and chronic seizures in mouse models [9]. Glutamate is a major neuromediator of the CNS and its effect on the influx of Ca^2+^ through NMDAR regulates enzyme activities and protein trafficking required for neuronal development and synaptic plasticity [10]. NMDAR is also a crucial effector in acute and chronic neurological diseases through glutamate excitotoxicity caused by intracellular Ca^2+^ overloading [10], [11]. Moreover, NMDAR has been shown to operate outside the CNS in bone, heart, lung, skin, and several endocrine and hematopoietic cells [12], [13]. On the basis of these observations, here we have explored the effect of HMGB1 on nerve terminals isolated from rat hippocampus and on human neuroblastoma cells expressing functional NMDARs [14], [15] and stimulated with sub-optimal concentrations of NMDA.

The rat hippocampal glutamatergic nerve terminals are endowed with NMDA receptors that respond to relatively low concentrations of NMDA [16]. Isolated purified nerve terminals (synaptosomes) prepared from rodent brain regions provide a helpful model allowing pharmacological characterization of the receptors sited on the plasmamembrane of the nerve terminals, and revealing direct effects of agents or modulators at the receptors themselves. In fact, release monitoring from a superfused synaptosomal monolayer [17], by removing any released compound and minimizing metabolism avoids receptor biophase and prevents indirect effects, enabling “nude” receptors to be exposed. In these conditions, when monitoring glutamate release, only targets located on glutamatergic nerve terminals are selectively acted upon, allowing to assess if a substance, added to the superfusion medium could directly interfere with the activation of the receptors under investigation. Presynaptic synaptosomes loaded with [^3^H]D-aspartate have been shown previously to undergo a NMDA concentration-dependent release of the unmetabolizable glutamate analogue [16]. Hence, synaptosomes are a well characterized system to assay the possible effects of HMGB1 on NMDAR by using the superfusion technique. An alternative cell model that we have selected to investigate the possible functional effects of HMGB1 on NMDAR is the human neuroblastoma SK-N-BE cell line. We have shown previously that following stimulation with NMDA these cells undergo activation of a Ca^2+^-dependent cascade, involving a calpain-mediated conversion of inactive nNOS to a soluble active enzyme, resulting in an increased cell production of NO [18].

Finally, as a non-nervous cell model, here we have utilized the erythroleukemia cell line described above on the basis of its known calcium-dependent and RAGE-independent activation of the differentiation program promoted by sub-nanomolar amounts of extracellular HMGB1 [19], [8]. Moreover, we have demonstrated in a previous report that these cells are able to process extracellular HMGB1 by limited proteolysis, producing a peptide that maintains the differentiation stimulatory activity of the whole protein [20]. Interestingly, this bioactive peptide is localized between the TLR4 and the RAGE binding sites identified in the B box of HMGB1 [21], [22] and doesn’t overlap with them. We have also investigated the possible role of this peptide in the modulation of HMGB1-activated calcium influx and in the subsequent cell responses.

The results obtained demonstrate that HMGB1 operates as a positive modulator of NMDAR reducing the amount of agonist necessary to obtain activation of the channel.

## Materials and Methods

### Reagents and Antibodies

[^3^H]D-aspartate (specific activity: 11.3 Ci/mmol) was from Amersham Radiochemical Centre (Buckinghamshire, UK); N-methyl-d-aspartate (NMDA), dizocilpine (MK-801), ifenprodil hemitartrate and *cis*-4-[Phosphomethyl]-piperidine-2-carboxylic acid (CGS 19755) were from Tocris Bioscience (Bristol, UK). Leupeptin, aprotinin, Pefabloc^®^ SC, 4,5-diamino-fluorescein diacetate (DAF-2DA), N-nitro-L-arginine methyl ester (L-NAME), calpain inhibitor-1, toluidine blue, Neutral Red Solution, Tween^®^ 20, Triton^®^ X-100, RPMI 1640, Basal Medium Eagle (BME), anti-β-actin antibody, glycine and all the salts and other reagents were from Sigma-Aldrich (Milan, Italy). Amersham ECL Advance™ Western Blotting Detection Kit and Protein A-sepharose were from VWR International PBI (Milan, Italy). Fetal bovine serum (FBS) was from Euroclone (Milan, Italy). Sodium deoxycholate was from Merck (Milan, Italy). Calcium Green™-1, AM was from Invitrogen (Milan, Italy). HMGB1_(130–139)_ peptide was from TIB Molbiol (Genova, Italy). Eukaryotic recombinant HMGB1 was obtained as described [23]. Contaminant amino acids in purified HMGB1, evaluated by assaying the trichloroacetic acid soluble concentration of αNH_2_ groups, were ≤1 nM in the experimental conditions used. Anti-nNOS antibody was from BD Transduction Laboratories (Milan, Italy); anti-NMDA Receptor subunit 1 (NR1; GluN1) and subunit 2 A/B (NR2A/B; GluN2A/B) antibodies were from Millipore S.p.A. (Milan, Italy), and anti-NMDA Receptor subunit 2B (NR2B; GluN2B) was from BD Biosciences (Milan, Italy), respectively. Peroxidase-conjugated anti-rabbit and anti-mouse antibodies were from Cell Signaling Technology (Celbio S.p.A, Milan, Italy); anti-HMGB1 antibody was from Abcam plc (Cambridge, UK).

### Animals

Adult male rats (Sprague-Dawley 200–250 g) were housed at constant temperature (22±1°C) and relative humidity (50%) under a regular light-dark schedule (lights on 7 AM-7 PM). Food and water were freely available. Experimental procedures and animal care complied with the European Communities Council Directive of 24 November 1986 (86/609/EEC) and were approved by the Italian Ministry of Health in accordance with Decreto Ministeriale 116/1992 (protocol number 29823–6 of 09 December 2010). All efforts were made to minimize the number of animals used and their suffering.

### Preparation of Purified Synaptosomes

After decapitation, the hippocampus was rapidly removed and placed in ice-cold medium; purified synaptosomes were prepared according to [24]. Purified synaptosomes obtained from rat brain were proven positive for the neuronal markers synaptophysin and MAP-2, and negative for the glial, microglial or oligodendrocyte markers GFAP, integrin-α-M or RIP, indicating negligible contamination by non-neuronal cells [25], [26]. Briefly, the tissue was homogenized in 10 volumes of 0.32 M sucrose, buffered at pH 7.4 with Tris-HCl, using a glass-Teflon tissue grinder (clearance 0.25 mm). The homogenate was centrifuged (5 min, 1,000 g at 4°C) to remove nuclei and debris, and the supernatant was gently stratified on a discontinuous Percoll gradient (2%, 6%, 10%, and 20% v/v in Tris-buffered sucrose) and centrifuged at 33,500 g for 5 min. The layer between 10% and 20% Percoll was collected and washed by centrifugation. The synaptosomal fraction obtained from rodent hippocampus has been previously proven positive for the neuronal markers MAP-2 and only scantily contaminated by the glial markers GFAP; biochemical and functional data both supported that the synaptosomal fraction was largely free from astrocyte processes or non-neuronal cells [27]. For release experiments, synaptosomes were then suspended in standard medium with the following composition (mM): NaCl 128, KCl 2.4, MgSO_4_ 1.2, CaCl_2_ 1.2, KH_2_PO_4_ 1.2, HEPES 10, glucose 10 (pH 7.3–7.4).

### Synaptosome Superfusion Experiments

Synaptosomes were incubated (15 min at 37°C) with [^3^H]D-aspartate (0.03 µmol/L), transferred to parallel superfusion chambers at 37°C and superfused (0.5 ml/min) with standard medium, as previously described [25]; starting from t = 20 min of superfusion, glycine (1 µM) was continuously present to allow NMDA autoreceptor activation in the presence of physiological concentration of Mg^2+^ ions (see [16]). Briefly, after 33 min superfusion, superfusate fractions were collected in 3 min samples (from the first fraction, basal1, B1 to B5); after 38 min of superfusion, synaptosomes were exposed (12 min) to NMDA. The effects of HMGB1 or HMGB1_(130–139)_ peptide, or of the NMDA receptor antagonists MK-801, CGS 19755 or ifenprodil, was evaluated by adding the drug 8 min before NMDA. At the end of superfusion, the radioactivity in synaptosomes and superfusate samples was determined by liquid scintillation counting. The efflux of radioactivity in each fraction was calculated as a percentage of the total radioactivity present at the onset of the fraction considered (fractional release). The mean tritium fractional release in the first two basal fractions was taken as the 100% control value for each chamber; tritium efflux was evaluated as the percentage variation of tritium fractional release with respect to the corresponding control value. The drug-evoked tritium efflux was measured by subtracting the area under the curve of percentage variations in tritium fractional release in appropriate control chambers from the area under the curve of the percentage variations in drug-treated chambers. In each experiment, at least one chamber was used as a control for each condition. Drugs were dissolved in distilled water or in physiological medium.

### Cell Culture

SK-N-BE human neuroblastoma cells (Interlab Cell Line Collection, ICLC, HTL96015, Italy) were cultured in RPMI 1640 containing 10% FBS, 10 U/mL penicillin, 100 µg/mL streptomycin and 4 mM L-glutamine. C44 murine erythroleukaemia (MEL) cells were cultured as described previously and induced to differentiate by adding 5 mM HMBA to a culture containing 10^5^ cells/ml [28]. The absence of mycoplasma contamination was established by routine assay with Venor®GeM (Minerva Biolabs, Milan, Italy). Cell proliferation and viability were evaluated by NRU assay [29].

### NO Assay

NO was measured using HEPES buffer with the following composition (mM): HEPES 10, NaCl 140, KCl 5, glucose 5, MgCl_2_ 1, CaCl_2_ 2, glycine 0.01, L-arginine 0.10, DAF-2DA 0.05 (pH 7.4) [18].

### Cell Apoptosis Assay

Cells were seeded at 7.5×10^5^/well in 6-well plates, grown for 24 h in BME (excitatory amino acids free) containing 2% FBS and then exposed to 100 µM NMDA, 500 µM NMDA, 500 pM HMGB1, or 100 µM NMDA in the presence of 500 pM HMGB1. As a positive control for apoptosis cells were exposed to 10 mM Ca^2+^. After 24 h, cells were lysed in 5 mM Tris buffer, containing 5 mM EDTA, 0.5% Triton X-100, pH 7.5, for 30 min. The lysates were centrifuged at 13,000 g for 20 min and the supernatants were subjected to electrophoresis in 1.5% agarose. DNA was visualized by ethidium bromide staining [30].

### Wound Repair Assay

Confluent cell monolayers were placed in BME (excitatory amino acids free) containing 2% FBS for 24 h. Scratch wounds were created with a sterile pipette tip, the suspended cells were removed and stimuli (100 µM NMDA, 500 µM NMDA, 500 pM HMGB1, or 100 µM NMDA in the presence of 500 pM HMGB1) were added. After 24 h, cells were fixed in 4% paraformaldehyde and stained with 0.1% toluidine blue. Wound closure efficiency was evaluated by the NIH ImageJ software.

### Evaluation of Neurite Outgrowth

5×10^5^ cells were exposed for 170 h to the following stimuli: 100 µM NMDA, 500 µM NMDA, 500 pM HMGB1, 500 pM HMGB1_(130–139)_, 100 µM NMDA in the presence of 500 pM HMGB1, or 100 µM NMDA in the presence of 500 pM HMGB1_(130–139)_. The proportion of neurite-bearing cells (processes longer than two diameters of the cell) was evaluated cells fixed and stained as specified above on five random fields of three independent experiments. The neurite length/cell diameter ratio was estimated using the NIH ImageJ software.

### Isolation of Detergent-soluble Membrane Preparations and Immunoprecipitation

The particulate fraction from C44 MEL cell lysates was obtained as described previously [31], whereas synaptosomes were lysed in 50 mM sodium borate buffer, 1 mM EDTA, 10 µg/mL aprotinin, 100 µg/mL leupeptin, 2 mM Pefabloc^®^ SC, pH 7.5 by three freeze-thaw cycles and briefly sonicated. Cell and synaptosome membrane fractions were obtained by centrifugation at 100,000 g for 15 min at 4°C, washed in 50 mM sodium borate buffer, 0.1 mM EDTA, pH 7.5, and solubilized in 50 mM sodium borate buffer, 0.1 mM EDTA, 1% sodium deoxycholate, pH 9.0 at 37°C for 60 min. After centrifugation at 100,000 g for 30 min at 4°C, the pH was adjusted to pH 8.0, and Triton^®^ X-100 was added to a final concentration of 0.1%. The detergent-soluble portion was dialyzed against 50 mM sodium borate buffer, 0.1 mM EDTA, 0.1% Triton^®^ X-100, pH 7.5 (IP buffer) by diafiltration using centrifugal filter devices (10 kDa cut-off) Amicon^®^ Ultra-4 (Millipore). After centrifugation at 100,000 g for 30 min at 4°C, 250 µg of soluble proteins were incubated for 4 h at 4°C with 200 ng of HMGB1. In competition experiments a 1000-fold molar excess of HMGB1_130–139_ peptide was also added. Before immunoprecipitation procedure, samples were pre-cleared for 60 min at 4°C using 30 µl of Protein A or G-sepharose diluted 1∶1 with IP buffer. Immunoprecipitation was carried out using 2 µg of anti-HMGB1 antibody or 1 µg of anti-GluN1 antibody [18]. Protein were separated by 8% (to detect NMDAR subunits) or 12% (to detect HMGB1) SDS-PAGE and subjected to Western blotting.

### [Ca^2+^]_i_ Assay

2×10^4^ cells were incubated in 200 µl of HEPES buffer containing 10 µM Calcium Green™-1, AM. After 40 min at 37°C cells were washed with HEPES buffer and HMGB1 and/or NMDA were added in 100 µl of HEPES buffer. The fluorescence intensity (Excitation 485 nm; Emission 535 nm) was measured before (F0) and 3 min after the addition of stimuli (F) using the top reading mode in the fluorescence multilabel reader LB 940 Mithras (Berthold Italia). Variations of the fluorescence values were calculated as the difference between F and F0.

### Western Blotting

The nitrocellulose membranes were blocked in 5% nonfat dry milk, 0.1% Tween^®^ 20 and incubated 16 h at 4°C with a primary antibody: anti-nNOS (1∶2500), anti GluN1 (1∶1000), anti-GluN2B (1∶2000), anti-GluN2A/B (1∶1000), anti-β-actin (1∶5000), anti-HMGB1 (1∶2000). Peroxidase-conjugated secondary antibodies (1 h at 20°C) were: anti-rabbit (1∶4000) and anti-mouse (1∶5000). Immunoreactive signals were developed using ECL Advance™ Western Blotting Detection Kit, acquired using Chemi Doc XRS, and quantified by the Quantity One Image Software (Bio-Rad Laboratories).

**Figure 1:**
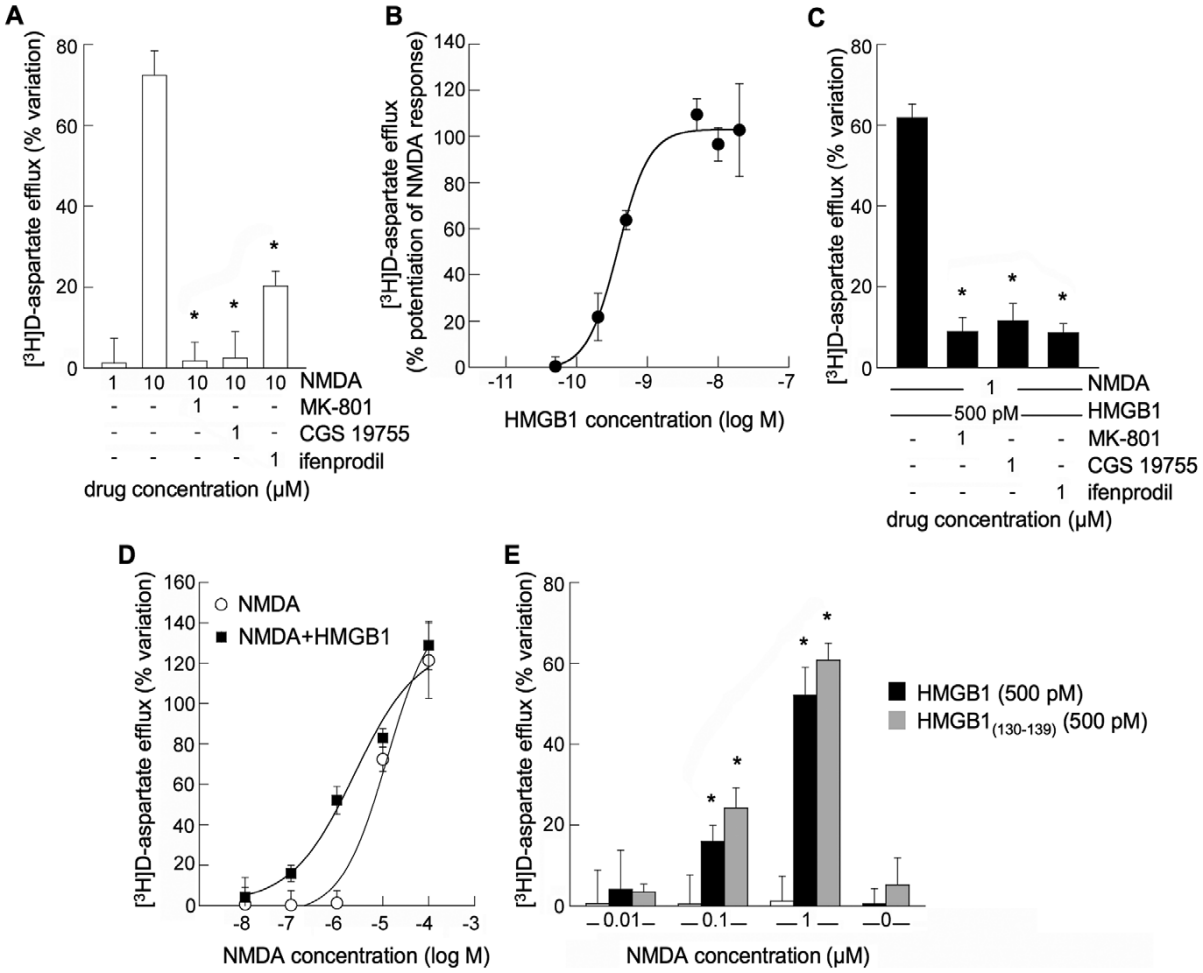
Effect of HMGB1 on presynaptic rat hippocampal NMDAR activation. (**A**) [^3^H]D-aspartate release from glutamatergic synaptosomes evoked by activation of NMDAR. Bars represent percent increase of [^3^H]D-aspartate release in the presence of the drugs. Data are means ± SEM of three to four independent experiments in triplicate. *p<0.05 when compared to the effect of the agonist alone. (**B**) Log concentration-response relationships for HMGB1 in evoking [^3^H]D-aspartate release from synaptosomes in the presence of 1 µM NMDA (per se ineffective, panel A). At the concentrations used HMGB1 was per se ineffective (data not shown). Data are means ± SEM of three to six independent experiments in triplicate. **(C)** Antagonism of HMGB1-evoked [^3^H]D-aspartate release in the presence of 1 µM NMDA. Bars represent percent increase of [^3^H]D-aspartate release in the presence of the drugs. Data are means ± SEM of three to four independent experiments in triplicate. *p<0.05 when compared to the effect of HMGB1 plus NMDA. (**D**) Log concentration-response relationships for NMDA-evoked release of [^3^H]D-aspartate from synaptosomes in the absence and in the presence of 500 pM HMGB1. Data are means ± SEM of three to six independent experiments in triplicate. (**E**) HMGB1_(130–139)_ mimics HMGB1 effect on presynaptic NMDAR. Bars represent percent increase of [^3^H]D-aspartate release from synaptosomes in the presence of the indicated additions. Data are means ± SEM of three to six independent experiments in triplicate. *p<0.05 when compared to the effect of NMDA alone.

### Calculation and Statistics

In the synaptosome superfusion experiments Log concentration-response relationship was obtained through a four parameter logistic function fitting routine (Sigma Plot software, Jandel-Scientific, San Rafael, CA, USA).

Significance of the difference was analyzed by ANOVA followed by post hoc Tukey’s test or, where indicated, by *t* test, using the Prism 4.0 software package (GraphPad Software, Inc, San Diego, CA), with statistical significance taken at p<0.05.

**Figure 2:**
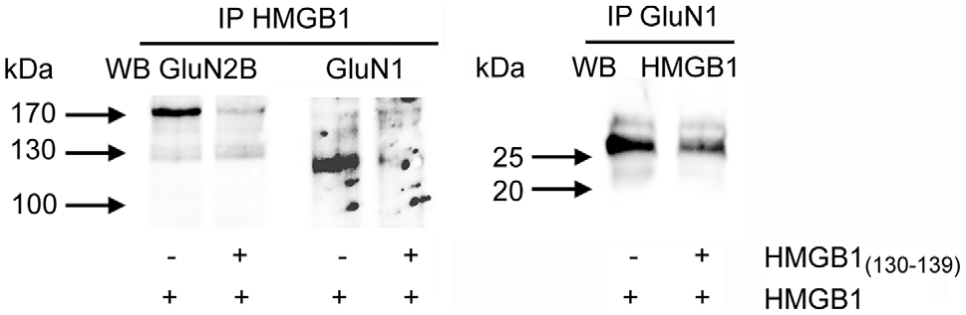
Identification of NMDAR/HMGB1 interaction. HMGB1, in the absence (−) or the presence (+) of HMGB1_(130–139)_, was added to solubilized membrane proteins from rat hippocampal synaptosomes. Immunoprecipitation procedure was performed using anti-HMGB1 (IP HMGB1) or anti-GluN1 (IP GluN1) antibodies. Immunoprecipitates were submitted to SDS-PAGE followed by Western blot analysis. A representative experiment (of three) is shown.

## Results

### Effect of HMGB1 on NMDA-evoked [^3^H]D-aspartate Release from Synaptosomes

Rat hippocampal synaptosomes loaded with [^3^H]D-aspartate were analysed for the efflux of the glutamate analogue in different conditions. The basal fractional tritium outflow in the first two fractions collected from superfused synaptosomes was 0.43±0.04%/min (n = 19). Addition of 10 µM NMDA, in the presence of 1 µM glycine, evoked [^3^H]D-aspartate efflux from superfused synaptosomes. This efflux was prevented by the NMDA channel blocker MK-801, by the NMDAR competitive antagonist CGS 19755 or by the non-competitive antagonist of GluN2B-containing NMDAR ifenprodil (Fig. 1A), indicating that activation of a NMDAR was responsible for this release. Addition of 1 µM NMDA in the presence of HMGB1 (0.2–40 nM), both per se ineffective when added separately, evoked a releasing response that was antagonized by MK-801, CGS 19755 or ifenprodil (Fig. 1B,C). In Fig. 1D, the shift to the left of the NMDA concentration-response curve in the presence of 500 pM HMGB1 is shown; a releasing response appeared at low-micromolar NMDA concentrations (0.1–1 µM), while the responses to high NMDA concentrations (10–100 µM) were unaffected. Since HMGB1 and HMGB1_(130–139)_ peptide have been shown previously to display similar RAGE-independent signaling activities [20] we have evaluated the effect of this HMGB1 fragment on synaptosomal [^3^H]D-aspartate efflux. As shown in Fig. 1E the HMGB1_(130–139)_ peptide mimicked the effect of HMGB1 on the synaptosomal responses to NMDA. HMGB1 or HMGB1_(130–139)_ (Fig. 1E) and MK-801, CGS 19755 or ifenprodil (data not shown) were per se ineffective on [^3^H]D-aspartate efflux from superfused synaptosomes.

These results indicate that HMGB1, used at a concentration close to that can be reached extracellularly *in vivo* [32], [33], lowers the concentration of NMDAR agonist required to obtain activation of the glutamate gated NMDA channel. Moreover, similar results can be obtained with a peptide of HMGB1 unable to activate both TLR4 and RAGE [21], [22]. Hence we can exclude that a cross-talk between these receptors is responsible for the HMGB1-dependent facilitation of NMDAR response to NMDA.

### Identification of NMDAR/HMGB1 Complex

To evaluate whether NMDAR and HMGB1 can undergo physical association we carried out an immunoprecipitation assay using recombinant HMGB1 and solubilized synaptosomal membranes. As shown in Fig. 2, GluN1 and GluN2B subunits (the former NR1 and NR2B subunits of NMDAR [34]) were found co-immunoprecipitated with HMGB1 using an immobilized anti-HMGB1 antibody directed towards the C-terminal region of HMGB1 (aminoacid residues from 150 to 215). Furthermore, immunoprecipitation assay performed using anti GluN1 antibody revealed that HMGB1 co-immunoprecipitates with NMDAR subunits. The presence of a 1000-fold molar excess of the HMGB1_(130–139)_ peptide markedly reduced the immunoreactive signals in both immunoprecipitation conditions. These results support the hypothesis that the potentiation of synaptosomal NMDAR, triggered by HMGB1 and by the HMGB1_(130–139)_ peptide at low concentrations of NMDA, is mediated by the interaction of this membrane ionotropic receptor with a specific region of HMGB1 localized in the B box upstream from the RAGE and downstream from the TLR4 binding sites.

### Effect of HMGB1 on Short-term Cell Responses to NMDAR Stimulation

**Figure 3:**
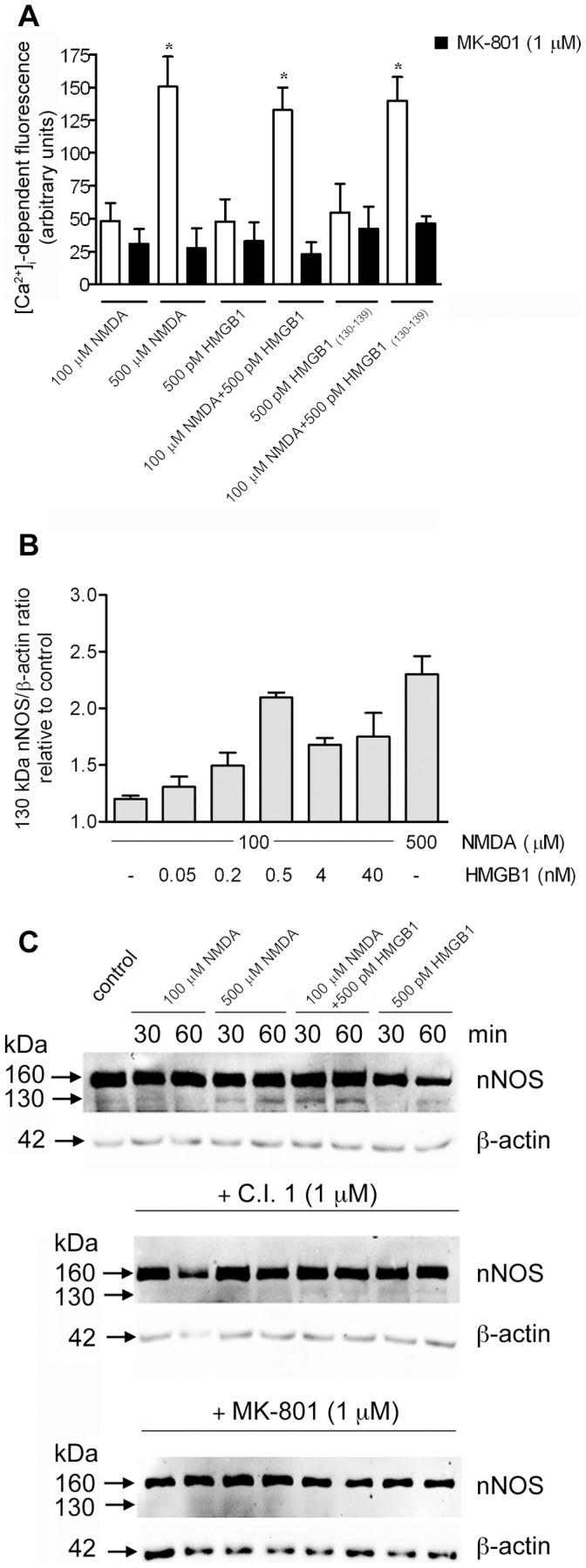
[Ca^2+^]_i_ elevation and activation of nNOS in SK-N-BE cells treated with NMDA and HMGB1. **(A)** Calcium Green™-loaded cells were exposed to the indicated stimuli. Data are means ± SD from three independent experiments in triplicate. *Significantly different from MK-801 treated cells (p<0.01, Tukey’s test). (**B and C**) 7×10^4^ cells were stimulated for 60 min (**B**) or for 30 and 60 min **(C)**, then subjected to Western blot analysis. (**B**) the levels of the 130 kDa nNOS form were calculated by densitometric analysis of the immunoreactive signals. Data are means ± SD of three different experiments. (**C**) Where indicated, cells were pre-treated with calpain inhibitor-1 (C.I. 1) or MK-801 for 30 min. Data are representative of three separate experiments.

We and others have demonstrated previously that SK-N-BE cells express a functional NMDAR calcium channel [14], [18]. To establish whether HMGB1 was able to promote a NMDAR-dependent increase of Ca^2+^-influx, cells were loaded with the permeant calcium fluorescent sensor Calcium Green™-1 AM and subjected to 180 s stimulation. As shown in Fig. 3A, cells exposed to 100 µM NMDA or to 500 pM HMGB1 alone did not show any appreciable elevation in Ca^2+^ influx compared to unstimulated cells, whereas 3-fold higher fluorescence was detectable in cells stimulated with 500 µM NMDA. This increase in [Ca^2+^]_i_ was prevented by the selective blocker MK-801 thus demonstrating its dependence on the opening of the NMDAR calcium channel [35]. However, cell stimulated with 100 µM NMDA in the presence of 500 pM HMGB1 showed an increased level of [Ca^2+^]_i_, comparable to that obtained with 500 µM NMDA. MK-801 abolished this cell response to the NMDA/HMGB1 co-stimuli demonstrating that HMGB1 facilitates the activation of NMDAR at concentrations of the NMDA agonist per se ineffective on these cells. A similar 3-fold increase in the [Ca^2+^]_i_-dependent fluorescence was obtained when cells were stimulated with 100 µM NMDA in the presence of 500 pM HMGB1_(130–139)_ peptide. Also in this condition MK-801 abolished the increase in cell calcium influx. This finding supports the evidences obtained with the synaptosomes, indicating that also on this neuroblastoma cell line the HMGB1_(130–139)_ peptide recognizes and activates NMDAR with an efficiency similar to that displayed by the whole HMGB1 molecule.

Thus, the association of HMGB1 to NMDAR through the HMGB1_(130–139)_ peptide lowers the amount of agonist required to activate the NMDAR channel-dependent rise of [Ca^2+^]_i_.

We have demonstrated previously that Ca^2+^-loading of SK-N-BE cells induced a calpain-mediated conversion of the 160 kDa inactive nNOS to a 130 kDa active synthase [18]. Here we have analyzed the possible functional role of HMGB1 on NMDAR-dependent activation of nNOS. As shown in Fig. 3B, the amount of 130 kDa nNOS form was not significantly different in unstimulated or 100 µM NMDA-treated cells, whereas a 2-fold increase was measured in cells stimulated with 500 µM NMDA. However, in the presence of 100 µM NMDA, HMGB1 concentration-dependently increased the production of 130 kDa nNOS with a peak response at 500 pM. At this concentration of HMGB1 the level of 130 kDa NOS was 90% of that obtained with optimal (500 µM) NMDA amounts. As shown in Fig. 3C, cell stimulation with 100 µM NMDA/500 pM HMGB1, from now on termed NMDA/HMGB1, resulted in a faster accumulation of the 130 kDa nNOS (maximum already at 30 min) compared to 500 µM NMDA (maximum at 60 min). HMGB1 alone at any concentration did not promote a significant change in the cell levels of 130 kDa nNOS (data not shown). This proteolytic activation of nNOS concerned only a very limited fraction of the native synthase, excluding a rapid cell depletion of the inactive enzyme. Both calpain inhibitor-1 (C.I. 1, Fig. 3C, middle panel) and the NMDAR blocker MK-801 (Fig. 3C, lower panel) prevented the production of active nNOS, regardless the stimulating conditions.

### Effect of HMGB1 on NMDAR Activated Cell Synthesis of NO

**Figure 4:**
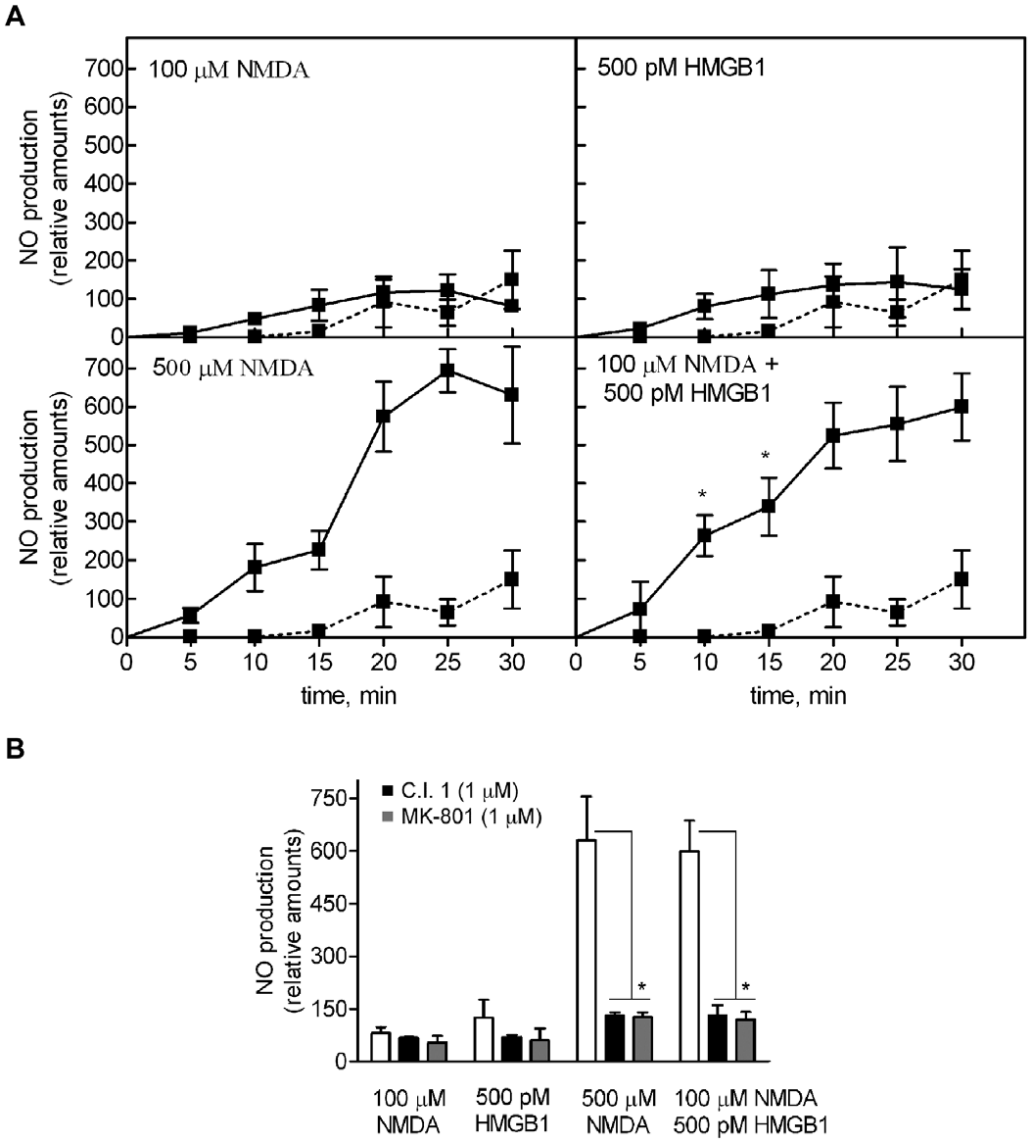
Synthesis of NO by SK-N-BE cells exposed to NMDA and HMGB1. DAF-2DA-loaded cells were stimulated with the indicated additions. (**A**) The kinetics of NO production was measured as the L-NAME-dependent increase in fluorescence (filled line). Basal cell production of NO was carried out in the absence of any addition (dotted line). Data are means ± SD of four different experiments in triplicate. * p<0.01 vs 500 µM NMDA-treated cells at the indicated times, according to *t* test. (**B**) C.I. 1 or MK-801 were added 30 min before the indicated stimuli. Data quantified at 30 min are means ± SD of four different experiments in triplicate. * Significantly different from indicated groups (p<0.01, Tukey’s test).

To assess the actual cell production of NO by cells stimulated with NMDA/HMGB1 we examined the level of NO in a time course analysis. As shown in Fig. 4A, cell exposed to 100 µM NMDA or to 500 pM HMGB1 did not produced detectable amounts of NO. Conversely, cell stimulated with 500 µM NMDA or with 100 µM NMDA in the presence of 500 pM HMGB1 synthesized NO, reaching similar levels of the radical at 30 min. However, at 10–15 min from the addition of the stimuli the concentration of NO was 1.5-fold higher in cells stimulated with NMDA/HMGB1 in comparison with 500 µM NMDA alone. This finding is consistent with the more rapid accumulation of active nNOS in cells exposed to NMDA/HMGB1 than to 500 µM NMDA (see Fig. 3C). Calpain inhibitor-1 and MK-801 abolished the NO synthesis in both stimulatory conditions (Fig. 4B). This result indicates that the accumulation of active nNOS promoted by HMGB1 at ineffective concentrations of the NMDAR agonist, is paralleled by a similar kinetics of NO increase, demonstrating the existence of a strict correlation between the level of 130 kDa nNOS form and NO production. Moreover, NMDA/HMGB1 induces a faster cell response compared with that obtained with optimal amounts of NMDA alone.

### Effect of HMGB1 on Long-term Cell Responses to NMDA Stimulation

**Figure 5:**
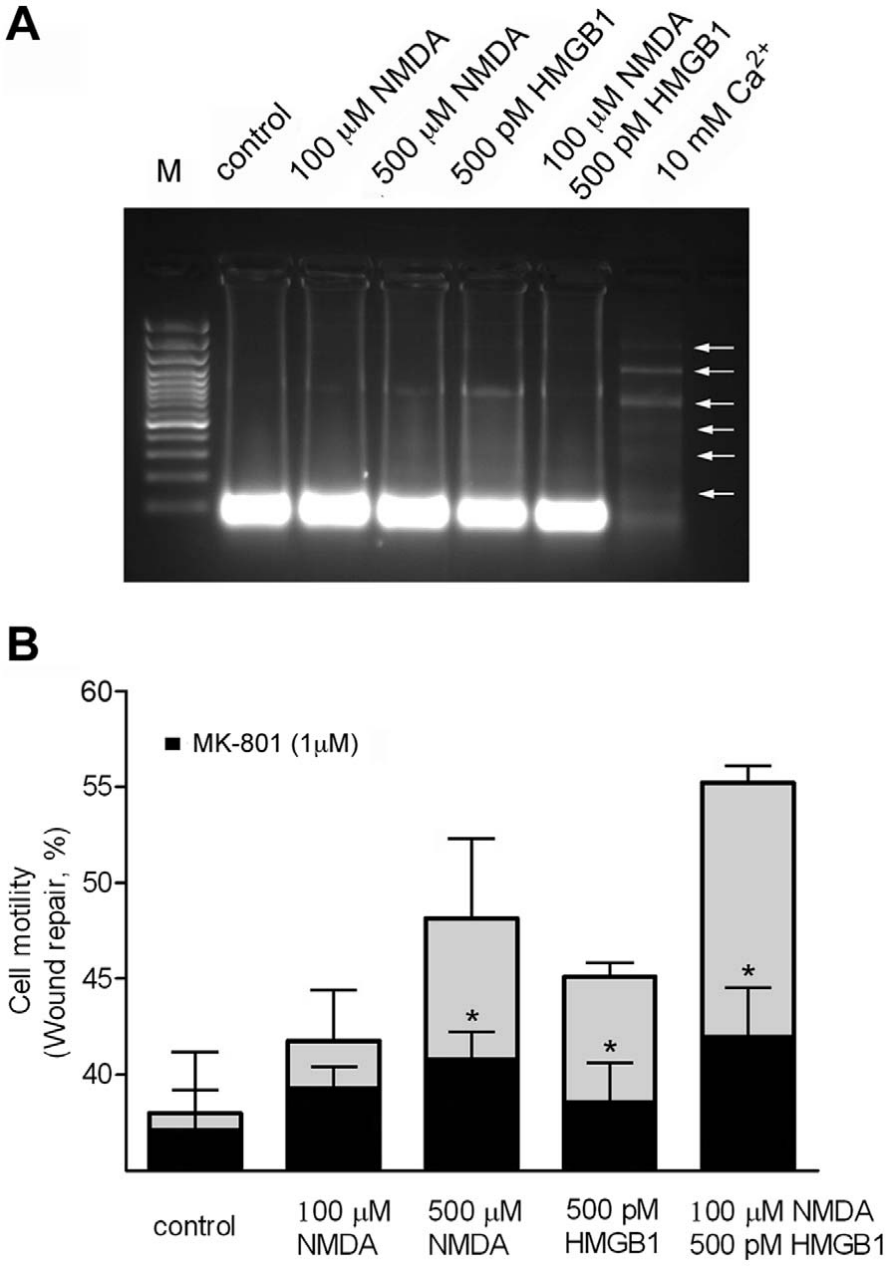
Effect of NMDA and HMGB1 on SK-N-BE cell death and motility. (**A**) Cell apoptosis was evaluated by measuring the appearance of nucleosomal DNA fragmentation after 24 h exposure to the indicated stimuli. M: 100bp molecular weight markers; control: vehicle-treated cells. The gel is representative of two experiments. (**B**) Wounded cell monolayers were treated with the indicated stimuli. MK-801 was added 30 min before cell stimulation. Data are means ± SD of three different experiments and expressed as percent of wound closure. * p<0.05 vs. cells treated with MK-801, according to *t* test.

It has been shown that cell motility requires [Ca^2+^]_i_ increase and calpain activation [36]. To establish whether the NMDA/HMGB1-dependent activation of calpain affects SK-N-BE cell motility, we carried out a wound repair assay. Since these analyses required prolonged cell exposure to the different stimuli we performed at first a Neutral Red Uptake assay (not shown) and a DNA fragmentation analysis (Fig. 5A) excluding that cell growth and cell death were significantly affected following 24 h cell exposure to the indicated stimuli.

The wound repair assay showed that maximal cell motility was triggered by cell co-stimulation with NMDA/HMGB1 (Fig. 5B). The significant stimulatory effect played by HMGB1 alone, but not observed by measuring cell calcium influx and NO production (see Fig. 3 and 4), could be attributed to the presence of excitatory amino acids in the fetal bovine serum present in the cell medium in these experimental conditions. In any case, cell pre-treatment with MK-801 almost completely prevented the enhancement of NMDA/HMGB1 cell motility (Fig. 5B), Interestingly, cell motility was also highly reduced by the NOS inhibitor L-NAME and the calpain inhibitor C.I.1 (not shown) indicating that all components of the calcium-activated cascade are fundamental to this cell activity.

**Figure 6:**
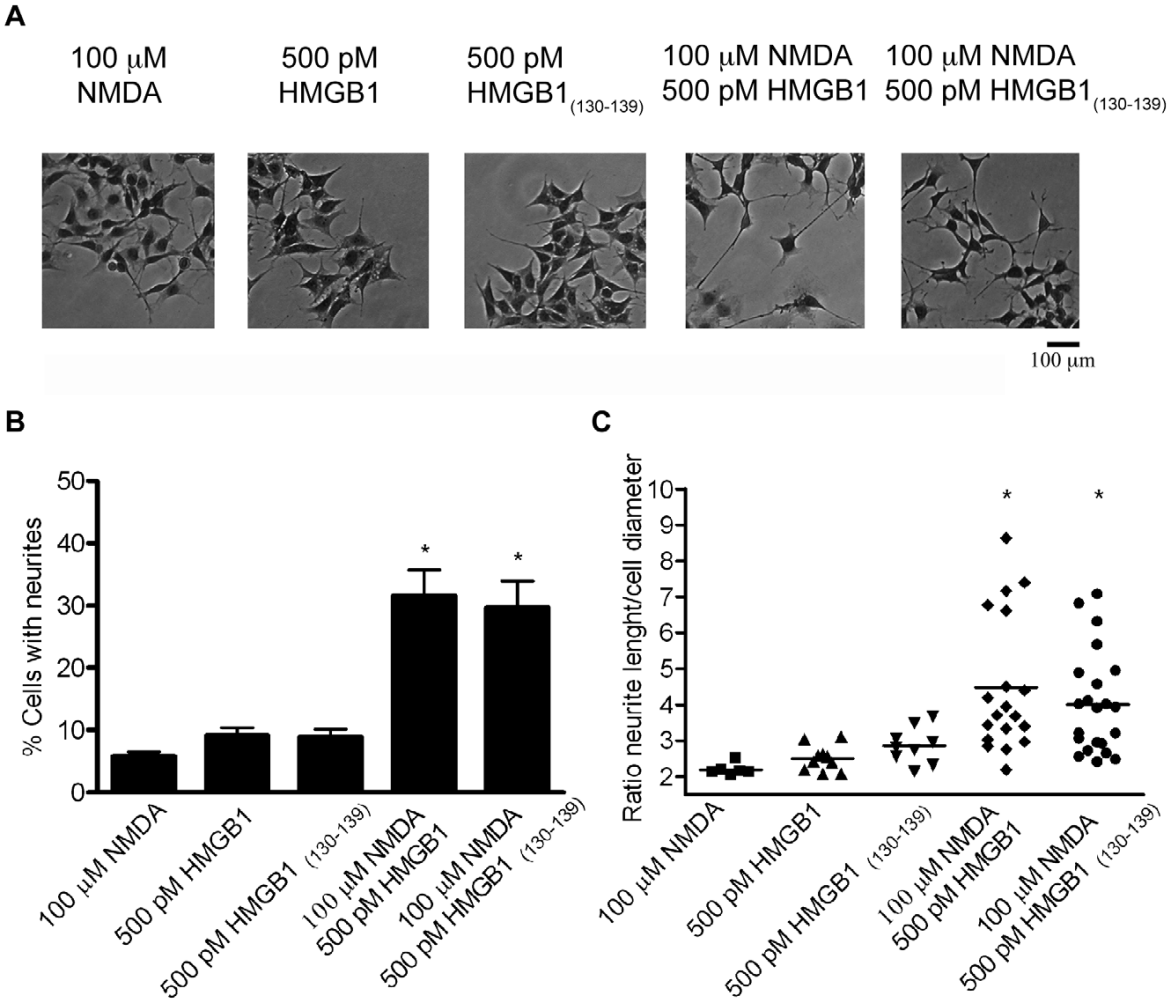
Neurite outgrowth of SK-N-BE cells stimulated with NMDA and HMGB1. (**A**) Representative images for each experimental condition are shown. (**B**) Proportions of neurite-bearing cells exposed to the indicated stimuli. Values represent the mean ± SD. * Significantly different from other groups (p<0.01, Tukey’s test). (**C**) Neurite length/cell diameter ratio of neurite-bearing cells. * Significantly different from other groups (p<0.05, Tukey’s test).

It has been shown previously that NOS activation is involved in neurite outgrowth of neuroblastoma cells [37]. Thus, we considered the NMDA-promoted neurite outgrowth as an additional experimental tool to explore the effect of HMGB1 on this process via NO production. As shown in Fig. 6A, the NMDA/HMGB1 co-stimulus increased the number of cells bearing neuritis. Specifically, NMDA and HMGB1 alone triggered neurite extensions only in 6±2% and 9±4% of the cells, whereas NMDA/HMGB1 co-stimulation induced this response in 32±12% of the cells (Fig. 6B). Moreover, the neurites extended in response to the combined stimuli were 1.8-2-fold longer than those of cells exposed to the single stimuli (Fig. 6C). Similar results were obtained with the HMGB1_(130–139)_ peptide utilized instead of full-length HMGB1. Thus, functional responses can be elicited in SK-N-BE cells by exposure to ineffective concentrations of excitatory amino acids in the presence of sub-nanomolar amounts of HMGB1.

### Involvement of NMDAR on HMGB1 Stimulated Erythroleukemia Cell Differentiation Induced by HMBA

**Figure 7:**
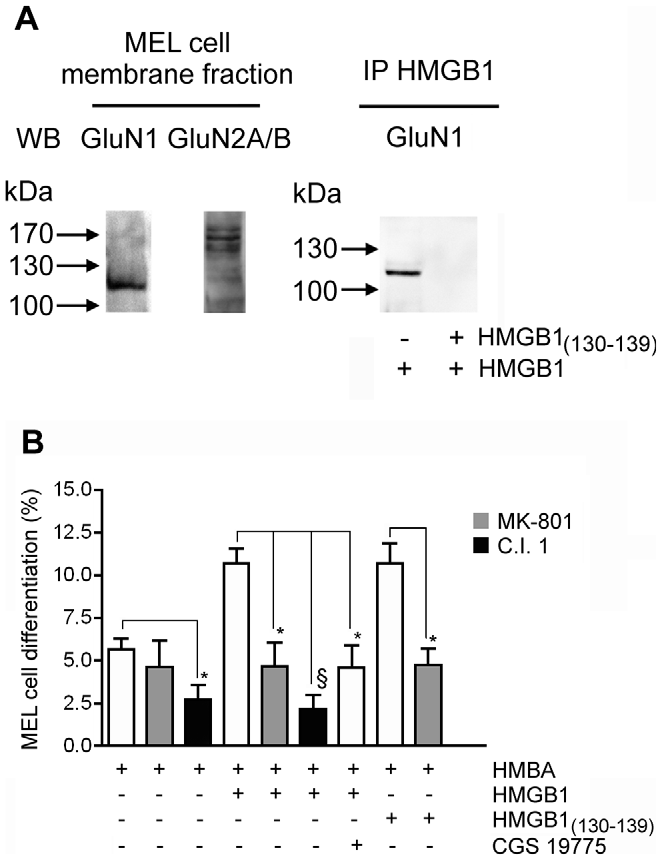
Involvement of NMDAR in HMGB1 promoted MEL cell differentiation. (**A**) 50 µg of solubilized membrane proteins from MEL cells were submitted to Western blot analysis. Immunoprecipitation of HMGB1 (IP) was carried out using solubilized MEL cell membrane proteins as specified in Materials and Methods. A representative experiment (of three) is shown. (**B**) HMBA-treated cells were exposed to the indicated additions (250 pM HMGB1, 1 µM C.I. 1, 1 µM MK-801, 50 µM CGS 19755). After 24 h the percentage of differentiated cells was evaluated by benzidine staining. Bars are means ± SD of four different experiments. *p<0.05, ^§^ p<0.01, vs cells stimulated in the absence of the indicated inhibitor, according to *t* test.

We demonstrated previously that MEL cell differentiation, induced by HMBA, is activated by extracellular HMGB1, independently of RAGE [8] and that the HMGB1_(130–139)_ peptide is endowed with an erythroid differentiation stimulatory efficiency similar to that shown by whole HMGB1 [20]. Since the onset of the MEL cell differentiation program requires an increase in intracellular Ca^2+^ concentration [19], here we evaluated whether NMDAR is involved as a mediator of HMGB1 signaling in these non-nervous cells. At first we assessed the presence of NMDAR on MEL cell solubilized membrane fraction. As shown in Fig. 7A, both GluN1 and GluN2A/B subunits were detectable. Hence, we determined whether NMDAR of MEL cell membranes co-immunoprecipitated with HMGB1 by measuring the presence of the GluN1 subunit in the immunoprecipitate. The GluN1 immunoreactive signal was detected in the HMGB1 immunoprecipitate but it was absent when a 1000-fold molar excess HMGB1_(130–139)_ peptide was added together with HMGB1. This result suggests that also in these erythroleukemia cells HMGB1 interacts with the NMDAR complex and that the HMGB1_(130–139)_ peptide competes with this binding.

Next we have explored the possible role of HMGB1/NMDAR on the differentiation process of erythroleukemia cells, induced by HMBA. As shown in Fig. 7B, after 24 h cell exposure to HMBA, 6% of MEL cells underwent erythroid differentiation and this value was not significantly affected by addition of the NMDAR blocker MK-801. In the concomitant presence of the HMBA/HMGB1 induction mixture, the proportion of differentiated cells increased to 12%, but this increase was abolished by addition of MK-801. This result suggests that NMDAR is a functional mediator of HMGB1-promoted differentiation in this MEL cell line. Moreover, cells induced with HMBA or with HMBA/HMGB1 mixture in the presence of 1 µM C.I.1 displayed a marked reduction of differentiation. It has been demonstrated that the C.I.1 at this concentration is a specific inhibitor of calpain [38]. Thus, calcium dependent proteolysis is required for the erythroid differentiation response induced by HMBA and enhanced by HMGB1.

MEL cells were routinely maintained in a culture medium containing glutamate, the natural agonist of NMDAR. Hence, we analysed the effect of CGS 19755, a selective competitive NMDAR antagonist, on HMGB1-promoted differentiation of MEL cells. As shown in Fig. 7B, CGS 19755 antagonized the stimulatory effect triggered by HMGB1 on MEL cell differentiation. This finding indicates that HMGB1 increases the rate of differentiation of MEL cell operating as a co-agonist of glutamate on NMDAR. As expected the HMGB1_(130–139)_ peptide enhanced MEL cell differentiation induced by HMBA showing an effectiveness similar to that identified for the whole HMGB1 protein. Addition of MK-801 inhibited this HMGB1_(130–139)_ peptide activity supporting the conclusion that this fragment of HMGB1 corresponds to the site involved in recognition and activation of NMDAR.

## Discussion

This study was aimed to identify the mediator of HMGB1 signaling operated through an increase of cell Ca^2+^ influx [6], [7]. Here we have demonstrated that the ionotropic glutamate-gated channel NMDAR is a specific cell target of extracellular HMGB1. The experimental evidences obtained in support of this conclusion are: 1) HMGB1 potentiates the activation of NMDAR on synaptosomes and cells of neuronal and non neuronal origin in the presence of sub-stimulatory amounts of agonist; 2) this co-stimulatory effect is mimicked by the HMGB1_(130–139)_ peptide; 3) HMGB1 co-immunoprecipitates with NMDAR; 4) this protein-protein interaction is prevented in the presence of the HMGB1_(130–139)_ peptide.

We have shown previously that HMGB1 alone was not able to induce the release of the glutamate analogue [^3^H]D-aspartate from hippocampal nerve terminals [27]. However, here we show that superfused hippocampal synaptosomes increased their responsiveness to NMDA in the presence of HMGB1. Specifically, HMGB1 promoted a significant release of [^3^H]D-aspartate already at 0.1 µM NMDA, a concentration of agonist more than one order of magnitude lower than that required to evoke the efflux of the neurotransmitter. A similar result was also obtained with the HMGB1_(130–139)_ peptide, a fragment of HMGB1 that we showed able to mimic HMGB1 signaling on MEL cells [20]. This effect played by HMGB1 was abolished by a noncompetitive (MK-801) and a competitive (CGS 19755) NMDA receptor antagonist, as well as by a negative allosteric modulator of GluN2B-containing NMDAR (ifenprodil). Effectiveness of the subunit-selective antagonist ifenprodil [39], is compatible with HMGB1 potentiating activation of GluN2B-containing NMDAR. The hypothesis is supported by co-immunoprecipitation of HMGB1 with GluN1 and GluN2B subunits of NMDAR.

This finding prompted us to define whether HMGB1 potentiates cell NMDAR activation at low agonist concentrations and the possible consequences on cell functions.

An early event promoted by HMGB1/NMDAR interaction in neuroblastoma cells is an increase in the level of [Ca^2+^]_i_ mediated by activation of the ionotropic receptor. This effect is sufficient to promote activation of calpain, which, on turn, converts the inactive nNOS into a 130 kDa active enzyme form that synthesizes NO. At a functional level, these HMGB1-dependent changes result in an increased neuroblastoma cell motility and neurite outgrowth, both processes known as positively affected by Ca^2+^, NO and HMGB1 [40]–[42]. By using selective inhibitors we have demonstrated that cell migration and neurite outgrowth promoted by HMGB1/NMDAR signaling must include increase in [Ca^2+^]_i_ as well as calpain activation and NO synthesis, because the inhibition of anyone of these processes is sufficient to impair the cell response.

Previous reports indicated that similar cell responses can be the result of HMGB1/RAGE interaction [41], [43]. However, in our experimental conditions HMGB1 is maximally effective at sub-nanomolar amounts, whereas the K_d_ of the HMGB1/RAGE complex is approximately 10 nM [44]. Moreover, the HMGB1_(130–139)_ peptide, that displays a NMDAR potentiation activity similar to that shown by the whole protein, is located upstream the sequence of HMGB1 identified previously as the region containing the RAGE binding motif (between the aminoacid residues 150 to 183 [44]). Thus, at least part of the cell responses observed using high amounts of immobilized HMGB1 could be promoted by a co-stimulation of RAGE and NMDAR, being glutamate present in those experimental conditions. Interestingly, nM concentrations of HMGB1 have been recently found able to inhibit L-type calcium channel in cardiomyocytes through a RAGE and TLR4-dependent signaling [45]. Here we have observed that maximal stimulation of NMDAR can be obtained with sub-nanomolar HMGB1. Hence, HMGB1 could operate as a modulator of the intracellular calcium concentration by alternative mechanisms depending on the identity of the receptors recognized on different cell types, on the affinity of HMGB1 for different receptors concomitantly expressed by single cells and on the local extracellular concentration of this cytokine-like molecule.

The NMDAR potentiating activity identified at concentrations of HMGB1 close to those locally reached in vivo [32], [33], acquires an important physiological significance for the different cell types expressing this glutamate ionotropic receptor. In fact HMGB1 lowers the amount of agonist required to obtain NMDAR activation both in the nerve endings, that are responsive at 10 micromolar concentration of agonist and in a cell line that requires fifty times higher amount of NMDA to activate the receptor. To date relatively little is known about the function of different NMDA receptor subtypes. In any case our present results suggest that HMGB1 can affect the behavior of cells expressing NMDAR also outside the synaptic environment and the CNS. As a non-nervous target of HMGB1 we have utilized an erythroleukemia cell line shown previously responsive to sub-nanomolar amounts of HMGB1 independently of RAGE [8]. We have now identified NMDAR as the cell target of HMGB1 involved in the activation of the erythroid differentiation of this cell line. Moreover, these cells are able to synthesize the HMGB1_(130–139)_ peptide, that maintains the differentiation-enhancing activity of the full length protein, by extracellular processing of HMGB1 [20]. The involvement of this HMGB1 fragment in the potentiation of NMDAR seems particularly important because, for the first time, a bioactive peptide obtained by cell limited proteolysis of HMGB1 has been shown to be endowed with signaling activity. The significance of this finding is evident considering that this peptide could be also locally produced in vivo redirecting HMGB1 from a multiple receptor activating protein to a specific cell surface target activator. Further investigation is required to demonstrate whether HMGB1 undergoes this extracellular modification in different pathophysiological conditions.

The present findings can explain on a molecular basis the mechanism by which the HMGB1-mediated effect can undergo a transition from a physiological to a pathological one. Our findings suggest that HMGB1 can facilitate GluN2B-containing NMDAR in nervous system. These receptors are often extrasynaptic and, compared with other NMDAR subtypes, appear to contribute preferentially to pathological processes linked to overexcitation of glutamatergic pathways (see [10], [46] and references therein). Accordingly, in addition to an increase in the level of glutamate, suggested to occur in neurodegeneration [47], we propose that these pathologic states could be worsened by elevation of extracellular HMGB1, in conditions of active and passive release of the cytokine [3]. As a consequence, an over-activation of Ca^2+^-dependent systems can lead to uncontrolled proteolysis and oxygen reactive species generation. On the basis of such considerations, the proposed mechanism could be considered as the target for new therapeutical approaches for pathologies involving glutamate excitotoxicity and dysregulation of Ca^2+^ homeostasis.
